# AGING IN PLACE: REVISITING OLD ASSUMPTIONS ABOUT WHAT PEOPLE WANT AS THEY GROW OLD

**DOI:** 10.1093/geroni/igz038.2236

**Published:** 2019-11-08

**Authors:** Marc A Cohen, Robyn Stone, Ruth Katz

**Affiliations:** 1 University of Massachusetts Boston Gerontology Institute, LeadingAge LTSS Center @UMass Boston, Boston, Massachusetts, United States; 2 LeadingAge, Washington, District of Columbia, United States

## Abstract

When people are asked how and where they would like to age, they overwhelmingly indicate they want to “age in place”, that is, in their own homes. To support this desire, a range of home and community-based service options have been developed accompanied with major declines in the use of nursing homes and other institutional services. However, what if we’ve been asking the wrong question? Or asking the wrong people? Given that upwards of 70% of people turning age 65 will have a need for long-term services and supports (LTSS) and 52% will have significant need, the more relevant question is: how might you want to age in the presence of LTSS needs? In this research we explore the attitudes and expectations of a nationally representative sample of 1,200 “late Boomers” age 60 to 72 (instead of all adults) regarding how they want their life to look should they become physically or cognitively impaired and need LTSS. The analytic sample was derived from NORC’s AmeriSpeak® Panel. We found that in the presence of significant LTSS need, many people -- 40% if physically disabled and 71% if cognitively impaired -- do not want to remain at home. More than anything else they value safety and do not want to burden their families. They are also concerned about feeling alone. Clearly, late boomers understand that there are circumstances where aging in place may not be right for them and implications for the way we invest resources in the service infrastructure.

